# Pharmacological inhibition of NF-κB prolongs lifespan of Drosophila melanogaster

**DOI:** 10.18632/aging.100314

**Published:** 2011-03-28

**Authors:** Alexey Moskalev, Mikhail Shaposhnikov

**Affiliations:** Institute of Biology, Komi Scientific Center, Ural Branch, Russian Academy of Sciences, Syktyvkar, Russia

**Keywords:** Drosophila melanogaster, NF-κB, aging, lifespan, anti-aging agents

## Abstract

Aging is associated with NF-κB-dependent pro-inflammation. Here we demonstrated that inhibition of NF-κB with pyrrolidine dithiocarbamate increases the median lifespan (13-20%) and the age of 90% mortality (11-14%) in Drosophila melanogaster females and males, respectively.

## INTRODUCTION

Aging is associated with diverse and widespread changes in gene expression in different animal species [1]. The NF-κB transcription factor is one of the major regulators of gene expression associated with mammalian aging [2-3]. NF-κB controls the expression of genes involved in innate immunity, inflammation and apoptosis [2,3]. Such age-dependent pathologies as tissue inflammation and atrophy are caused by over-activation of the NF-κB signaling with age [4-6]. We proposed that pharmacological inhibition of NF-κB will prevent age-related pathology and increase lifespan of *Drosophila melanogaster*. Three NF-κB transcription factors have been identified in *Drosophila*: Dorsal, Dif and Relish [7-9]. Like their vertebrate counterparts, these fly proteins contain an approximately 300-amino acid long, multifunctional and conserved Rel homology domain, which mediates dimerization, DNA binding, and interactions with IκB inhibitor proteins [7-9]. In this study we examined the effect of a specific NF-κB activity inhibitor, pyrrolidine dithiocarbamate (PDTC), on the lifespan of *Drosophila melanogaster*.

## RESULTS

**Figure 1.**
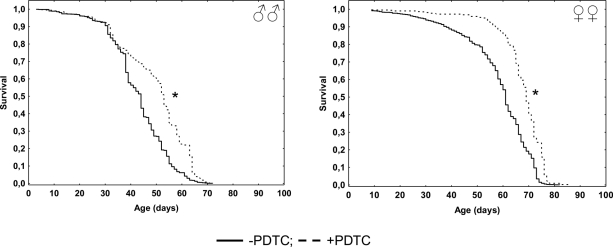
Effect of a NF-κB inhibitor (PDTC) on the survival functions * p<0.001 Kolmogorov-Smirnov test.

**Figure 2.**
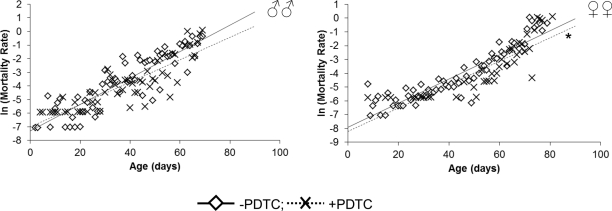
Effect of a NF-κB inhibitor (PDTC) on the rate of the age-dependent mortality * *p*<0.001 Maximum likelihood method.

In males pharmacological inhibition of NF-κB results in an increase of the median (by 20%) lifespan, as well as the age of 90 % mortality (by 14%), compared to males that did not receive PDTC treatment (Table, Figure 1). At the same time, the age-dependent mortality rate was not affected (χ^2^ = 0; df = 1) (Table, Figure 2). In females, pharmacological inhibition of the NF-κB transcription factor causes an increase in the median (by 13%) and maximum (by 11%) lifespan, as well as the age of 90 % mortality (by 6%), compared to females that did not receive PDTC treatment (Table, Figure 1). The inhibition of NF-κB factor increased the average and median lifespan (by 11-13%). The statistically significant decrease in the age-dependent mortality rate was detected in females as a result of NF-κB inhibitor effect (χ^2^ = 62.6; df = 1) (Table, Figure 2).

## DISCUSSION

Recent studies suggest that the NF-κB transcription factor controls age-dependent changes in inflammation genes expression. Donato *et al* showed that an increase of NF-κB dependent genes in human endothelium with age is primarily linked to decreased IκB-mediated NF-κB inhibition [10]. Age-associated expression of NF-κB-dependent genes cause progression of athero-sclerosis in rat [11]. Furthermore, selective inhibition of NF-κB activity in blood vessel endothelial cells prevents atherosclerosis progression [12]. Genetic blockade of NF-κB in the skin of chronologically aged mice reverses the global gene expression program and tissue characteristics to those of young mice [13,14]. Decrease of NF-κB activity impedes progression of degenerative phenotype in mice with knocked out *Sirt6* window sirtuin gene [15]. Kawahara *et al* suggested SIRT6 can prevent NF-κB-dependent gene overactivation via deacetylation of lysine 9 of the H3 histone (H3K9) on the promoters of NF-κB target genes, whereas over-activation of NF-κB promotes normal and accelerated aging [15]. However, the effect of NF-κB inhibition on the lifespan was not studied before. We have shown that pharmacological inhibition of NF-κB by pyrrolidine dithiocarbamate results in the considerable increase of longevity *Drosophila melanogaster*.

## METHODS

### *Drosophila melanogaster* strains

The experimental orphan flies'genotype was *y*.

### Pharmacological inhibition of NF-κB

Pyrrolidine dithiocarbamate (PDTC, Sigma) was used as a specific inhibitor of the NF-κB transcription factor [16]. The experimental flies where treated with yeast paste containing 20 mg/l PDTC throughout the lifetime. Control flies were fed with yeast paste without PDTC.

### Lifespan analysis

Control and experimental flies were maintained at 25±0.5°C in a 12 h-12 h light/dark cycle on a sugar-yeast medium [17] covered with the yeast paste. To estimate the longevity 150-200 flies were collected during 24 h since the onset of eclosion (about 30 adult flies per 120 ml vial) for each experimental variant. Males and non-virgin females were kept separately. Flies were transferred to a fresh medium three times a week. Dead flies were counted daily. For each experimental variant 2-4 biological replicates were pooled. Survival functions were estimated using the Kaplan-Meier procedure and plotted as survival curves. Mean, median, minimum, and maximum lifespan, the age of 90% mortality were calculated. For aging rate estimates, the natural logarithm of age-dependent mortality rate ln(*μ*(*x*)) were used to determine the *μ* and *R*_0_ parameters of the Gompertz equation (*μ*(*x*) = *R*_0_e*^μx^*) and the mortality rate doubling time (MRDT) was calculated using the formula MRDT = ln2/*μ* [18]. The statistical analysis of survival data was conducted using nonparametric methods. Comparison of survival functions was done using the modified Kolmogorov-Smirnov test [19]. The statistical significance of differences between the median life spans for the experimental and control variants was determined using the Gehan-Breslow-Wilcoxon [20] and Mantel-Cox tests [21]. To test the statistical significance of differ-ences in maximum lifespan (age of 90% mortality), the Wang-Allison test was used [22]. According Wang-Allison test all animals from two compared variants were combined and the 90th percentile of the lifespan was calculated. Then each animal in each experimental variant was categorized into one of two groups: either lifespan above the 90th percentile or lifespan below the 90th percentile. The 2×2 contingency table was used to record data. The ordinary χ2-test was used for an independent test of two experimental variants. Therefore, a test of the equality of proportions above the 90th percentile across the variants was used as a test of the equality of the percentiles across the two variants [22]. The significance of differences in age-dependent mortality rate and initial mortality rate (parameters *μ* and *R*_0_ in the Gompertz equation) was evaluated using the maximum likelihood method [23]. Statistical analysis was carried out using *Statistica* version 6.1, StatSoft, Inc. and *WinModest* version 1.0.2 [23] software.

**Table.** Longevity parameters of flies after pharmacological inhibition of the transcription factor NF-κB activity by PDTC

Variant	Sex	$\bar{x}\pm \Delta m$	*M*	*90 %*	*Min*	*Max*	*MRDT*	*α*	*R_0_*	*n*
−PDTC	Male	42.8±0.3	44	56	3	72	7.3	0.095	0.0010	1156
+PDTC		49.1±0.7	53*	64*	5	71	7.4	0.094	0.0005	381
−PDTC	Female	58±0.4	61	72	9	82	6.7	0.104	0.00014	1187
+PDTC		67.3±0.5	69*	76*	9	86	4.4	0.159*	0.0001	322

$\bar{x}\pm \Delta m$ mean lifespan and standard error of the mean; *M* – median lifespan; *90 %* – age of 90 % mortality; *Min* and *Max* – minimum and maximum lifespan; *α* and *R_0_* – Gompertz equation parameters; *MRDT* – mortality rate doubling time (ln2/α); *n* — sample size; +PDTC −PDTC treatment; −PDTC – without PDTC treatment.

* *p*<0.001.
